# The fast continuous wavelet transformation (fCWT) for real-time, high-quality, noise-resistant time–frequency analysis

**DOI:** 10.1038/s43588-021-00183-z

**Published:** 2022-01-27

**Authors:** Lukas P. A. Arts, Egon. L. van den Broek

**Affiliations:** Department of Information and Computing Sciences, Utrecht University, Utrecht, The Netherlands

**Keywords:** Software, Computational science, Computer science, Electroencephalography - EEG, Electrophysiology

## Abstract

The spectral analysis of signals is currently either dominated by the speed–accuracy trade-off or ignores a signal’s often non-stationary character. Here we introduce an open-source algorithm to calculate the fast continuous wavelet transform (fCWT). The parallel environment of fCWT separates scale-independent and scale-dependent operations, while utilizing optimized fast Fourier transforms that exploit downsampled wavelets. fCWT is benchmarked for speed against eight competitive algorithms, tested on noise resistance and validated on synthetic electroencephalography and in vivo extracellular local field potential data. fCWT is shown to have the accuracy of CWT, to have 100 times higher spectral resolution than algorithms equal in speed, to be 122 times and 34 times faster than the reference and fastest state-of-the-art implementations and we demonstrate its real-time performance, as confirmed by the real-time analysis ratio. fCWT provides an improved balance between speed and accuracy, which enables real-time, wide-band, high-quality, time–frequency analysis of non-stationary noisy signals.

## Abstract (web summary)

The authors present an open-source framework that enables fast and accurate time–frequency analysis of signals and demonstrate it on real-world applications, such as signals from the brain–computer interface.

## Main

**Fig. 1:**
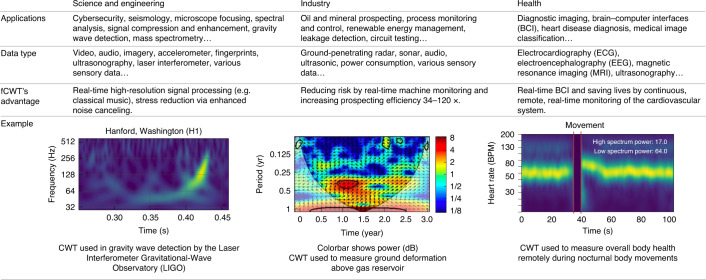
The impact of time–frequency analysis across society. In both nature and technology, signals enable communication, and processing techniques such as the CWT (also called IWT) are applied throughout. CWT was the primary processing method used in the Laser Interferometer Gravitational-wave Observatory (LIGO) experiment to detect gravity waves in highly non-stationary gravitational wave data. In industry, CWT has been applied to enhance mineral detection and speech segmentation. CWT also allows the detailed analysis of biosignals such as an electrocardiogram in the medical domain. BCI, brain–computer interface; BPM, beats per minute. Image credits: (left) adapted with permission from ref. ^82^, Caltech/MIT/LIGO Laboratory; (center) adapted from ref. ^83^ under a CC BY license.

Signals are essential in both nature and (man-made) technology, because they enable communication^1,2^ (Fig. 1). Mathematically, a signal is a function of one (for example, speech) or more (for example, a two-dimensional (2D) image) dimensions that carries information about the properties (for example, state) of a physical system^3^. A source transmits a signal via a channel to a receiver, which delivers it to its destination. For example, a brain sends an oral message via vocal cords through the air, which is received by the listener’s ear, which brings it to the listener’s brain. When the same message is transmitted via a smartphone, the air is complemented by a chain of technology, leaving the rest of the chain untouched. Signals are omnipresent in society^3,4^ (Fig. 1).

Independent of its source, a signal needs to be processed to enable the generation, transformation, extraction and interpretation of the information it is carrying^3^. A widely used method to interpret (that is, extract and analyze) repeating patterns in signals is the Fourier transform (FT)^3,4^. A FT transforms a function of time into a complex-valued function of frequency, representing the magnitudes of the frequencies. The FT assumes the signal is stationary. In other words, it is a stochastic process in which the marginal and joint density functions do not depend on the choice of time origin^2^. However, in real-world practice, this assumption is often violated. Consequently, the FT is unable to process real-world non-stationary signals reliably^5^. To circumvent the problem of non-stationarity, advanced algorithms exist that analyze a signal based on their decomposition in elementary signals that are well localized (or boxed) in time and frequency^4^. These include the short-term Fourier transform (STFT), also known as the Gabor transform, and the wavelet transform (WT)^6^.

The STFT is very similar to the FT, but it uses a window function and short wavelets localized in both time and frequency, instead of pure waves, to extract temporal and spectral information. The drawback of the STFT is its use of a fixed-width window function, as a result of which frequency analysis is restricted to frequencies with a wavelength close to the window width^7^. Additionally, chopping up the signal in short, fixed-width windows scrambles the signal’s properties. Accordingly, the frequency analysis is affected^8^.

The WT overcomes the drawback of the STFT by not relying on a window function. Instead, it uses a family of base functions that dilate and contract with frequency to represent the signal, thereby ensuring high resolution across the entire frequency spectrum. Consequently, the WT suffers from a high computational load. This prohibits its use with low-end hardware and for real-time applications^9^, as real-time computation requires an algorithmic computation time that is smaller than the signal’s duration.

**Fig. 2:**
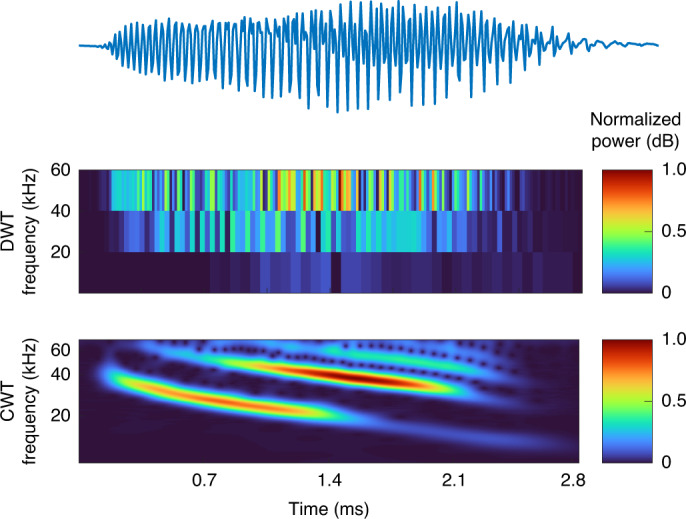
Comparison of DWT and CWT. A time-varying pulse signal of a sonar device is analyzed in the range 0–60 kHz using the DWT and the CWT. The DWT uses a coarse time–frequency discretization to favor speed. By contrast, the CWT uses a time-consuming near-continuous discretization of the time and frequency scales to favor resolution. Source data

To reduce the computational burden of the WT, the discrete wavelet transform (DWT) has been proposed, which applies a coarse, logarithmic discretization. This makes DWT suitable for data compression, but simultaneously disqualifies it from use in detailed analysis, as it is not able to analyze intricate time–frequency details^8^ (as shown in Fig. 2). For this, a true WT—the computationally expensive continuous wavelet transform (CWT)—also called an integral wavelet transform (IWT), is needed. CWT offers a high-resolution representation of the time–frequency domain by using near-continuous discretization. Its continuous time and frequency scales better support intricate time–frequency analysis. Consequently, CWT is often described as the mathematical microscope of data analysis^10^ (Fig. 2).

In this Resource paper we introduce the open-source fast continuous wavelet transform (fCWT), which brings real-time, high-resolution CWT to real-world practice (for example, biosignals^11–13^, cybersecurity^14,15^ and renewable energy management^16,17^; Fig. 1). Next, we assess the performance of fCWT in a benchmark study and then validate the use of fCWT on synthetic, electroencephalography (EEG) and in vivo electrophysiological data. We end with a concise discussion.

## Results

The performance of fCWT was benchmarked against six widely used CWT implementations, then it was subjected to a threefold validation on accuracy, resolution and throughput using, respectively, synthetic data, human EEG data and high-density in vivo extracellular rodent electrophysiology.

### Benchmark

**Fig. 3:**
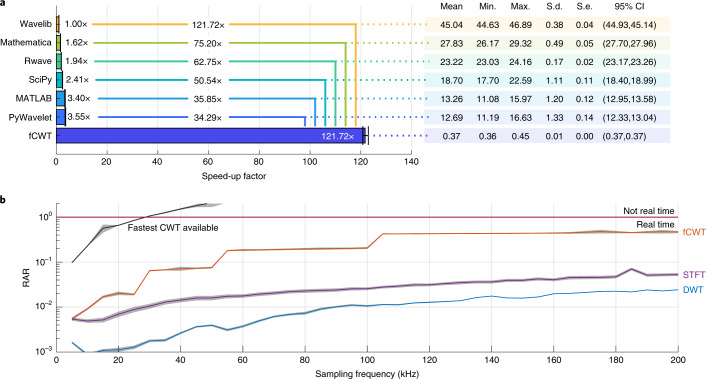
Benchmarking with fCWT and six state-of-the-art time–frequency methods. **a**, The average speed-up of fCWT and six publicly available implementations after 100 runs on a signal of length *N* = 100,000 with accompanying statistics (in seconds). The signal was analyzed using 3,000 frequencies ranging from *f*_0_ = 1 Hz to *f*_1_ = 32 Hz. **b**, The RAR (equation (1)) of fCWT (600 frequencies, *σ* = 6), the fastest CWT available (PyWavelet’s CWT, 600 frequencies, *σ* = 6), STFT (500-ms Blackman with 400-ms overlap) and DWT (four-order Debauchie 20 levels) versus sampling frequency on a 10-s synthetic signal. Parameters were chosen to reflect actual usage in real-world applications. Jumps in the performance of fCWT are explained in the Methods. Source data

To benchmark the performance of fCWT we compared fCWT to the six widely used CWT implementations shown in Fig. 3. Because of its widespread use across research, the complex Morlet wavelet (*σ* = 6) was used to calculate the CWT of three signals, all containing *N* = 100,000 samples. The Morlet wavelet is defined as a plane wave modulated by a Gaussian envelope. The parameter *σ* controls the time–frequency resolution trade-off^18^. The first signal was generated to be non-stationary using a sine wave whose frequency changed linearly from *f*_start_ = 1 Hz to *f*_end_ = 7 Hz. The second and third signals contained uniformly random noise and a stationary piecewise defined function, respectively. Three different signals were used to prove fCWT’s flexibility and signal independence. Nevertheless, the signal content and wavelet choice are irrelevant to the performance of fCWT (see Methods for details).

All CWT implementations, including fCWT, use a near-continuous frequency scale containing 3,000 frequencies (range, *f*_0_ = 1 Hz to *f*_1_ = 32 Hz), evenly spaced in exponential space. fCWT thus features a high-frequency resolution in the low-frequency spectrum and a lower frequency resolution in the high-frequency spectrum.

PyWavelet^19^ and SciPy^20^ execution times were measured in a Python 3.8.6 environment, using the Timeit library inside the code to exclude compile time. The overhead resulting from the translation between C and Python was removed by estimating the intersection factor of the linear relationship between signal size and execution time. MATLAB v2019b and Mathematica 12.0.0.0 execution times were measured using the program-specific timing functions that measure the exact kernel execution times.

Wavelib^21^ was used as the benchmark’s baseline algorithm as it is the reference CWT C/C++ library^9^, and most microcontrollers are programmed using C/C++. Wavelib^21^ thus serves as a baseline for the reported speed-ups (Fig. 3). The reported execution times were obtained from an eight-core 2.30-GHz central processing unit (CPU) via 100 successive runs, which removed the influence of caching behavior. A 10-s pause between runs was implemented to prevent the CPU from overheating. Outliers that deviated by more than 3 s.d. from the mean were removed. Wavelib and SciPy had three outliers, leaving *N* = 97 samples for all algorithms to ensure equal group sizes. A repeated-measures analysis of variance (ANOVA) revealed that the algorithms differed significantly, *F*(4, 93) = 2,474,778.911, *P* ≪ 0.001, *η*^2^ = 1.000, where *F* denotes the ANOVA statistic based on the ratio of mean squares, which indicates the ratio between the explained and unexplained variance or, in other words, the between- and within-group variability. *P* is the probability that an observed difference occurred by chance, and *η*^2^ ‘indicates the proportion of variance accounted for (that is, a generalization of *r*/*r*^2^ and *R*/*R*^2^ in correlation/regression analysis)^13^. Also, all pairwise comparisons were highly significant (*P* ≪ 0.001, Bonferroni-corrected), with fCWT being, respectively, 122 times and 34 times faster than the reference Wavelib^21^ and the fastest available algorithm, PyWavelet^19^. Figure 3 presents descriptive statistics for all distributions.

The fast running time of fCWT was also compared to two other fast time–frequency estimation algorithms: the STFT and DWT. In this benchmark, STFT uses a Blackman window of 500 ms with 400-ms overlap, and DWT uses 20 dyadic (that is, *a*^*j*^ = 2^*j*^) scales of Debauchie decomposition. The parameters were chosen to reflect actual usage in real-world applications (Fig. 1). Both algorithms are implemented and benchmarked in MATLAB using the in-program timing functions. CWT implementations use 600 frequencies, evenly spaced in exponential space. Fewer frequencies are used to reduce memory usage.

To assess whether or not the algorithms perform in real time (that is, an algorithmic computation time less than the signal’s duration), we define the real-time analysis ratio (RAR):

1 $${{{\rm{RAR}}}}={\frac{{{\Delta }}{t}_{\rm{computation}}}{{{\Delta }}{t}_{\rm{signal}}}},$$

with Δ*t*_computation_ and Δ*t*_signal_ being the duration of the computation and signal, respectively. In the case of RAR > 1, an algorithm does not operate in real time. In the case of RAR just shy of 1, the algorithm is unlikely to run in real time as the time–frequency calculation is merely one step in a processing pipeline. When RAR ≪ 1, real-time operation is likely to be achieved or within reach. For all six CWT implementations and two traditional time–frequency techniques (that is, STFT and DWT), Fig. 3b shows RAR versus sampling frequency. The RARs were obtained by averaging 100 successive runs on 10-s signals with varying sampling frequencies (range, *f*_s0_ = 1 kHz to *f*_s1_ = 200 kHz). fCWT and CWT used 5-s signals to fit memory constraints. Small fluctuations in RAR are caused by the stochastic nature of benchmarks performed under real-world conditions. It should be noted that the sampling frequency is directly related to the number of samples. Therefore, we test fCWT’s performance for different signal lengths.

STFT and DWT exhibit superior real-time behavior on signals with sampling frequencies up to 200 kHz and beyond. However, they achieve these very high speeds because of their considerable drop in precision, as shown in Fig. 2. Therefore, STFT and DWT are not suitable for wide-band high-resolution time–frequency estimation. In these cases, CWT is favored. However, even the fastest CWT implementation available tends to be extremely slow compared to STFT and DWT. fCWT merges the best of both worlds, yielding real-time behavior on signals with sampling frequencies up to 200 kHz. This has brought CWT’s execution time close to that of STFT and DWT, while having 25 times to 100 times the spectral resolution of DWT throughout the spectral domain. As such, fCWT is a truly competitive real-time, high-resolution alternative for STFT and DWT.

fCWT allows signals with 34 to 122 times the sampling frequency of existing CWT implementations. Figure 3 shows fCWT’s capability of analyzing signals up to 200 kHz in real time, whereas the fastest implementation of CWT fails at *f*_s_ = 30 kHz. Consequently, fCWT enables real-time analysis of high-frequency signal dynamics, as exist in audio (for example, loudspeaker characterization^22^, full band speech coding^23^ and paralinguistic analysis^24^), biosignals (for example, brain–computer interfaces^12^ and peripheral signals such as ECG, electromyography, electrodermal activity and respiration^11,13^), image and video (for example, distance transforms^25,26^), sonar and radar^27,28^, network analysis (for example, renewable energy management^16,17^ and cybersecurity^14,15^) and machine fault diagnosis^29,30^ (Fig. 1).

### Synthetic data

fCWT’s spectral resolution is equal to that of CWT. In contrast to many other CWT optimization studies, we do not compromise precision. To demonstrate this, we compared fCWT to CWT on both clean and noisy synthetic datasets (see Data availability statement for details). Each dataset consists of three wavepackets that validate an algorithm on spectral and temporal resolution and bandwidth size. A noisy dataset was generated to mimic realistic conditions and assess noise resilience.

Quantitative assessment of each algorithm’s performance is carried out by calculating the per-wavepacket mean absolute percentage error (MAPE) scores of 100 runs on both datasets between actual frequencies and the time–frequency ridges extracted from the spectra (see Methods for details). The MAPE scores of the clean data are based on one run, as they are completely deterministic. We used a relative error measure to weight errors at all frequencies evenly.

Next to fCWT and CWT, STFT and DWT were also included, allowing us to show the speed–accuracy trade-off that currently dominates the time–frequency landscape. STFT is based on calculating multiple traditional FTs with overlapping fixed-sized windows. The STFT is very fast and efficient as it relies on the fast Fourier transform (FFT). However, the use of fixed-sized windows requires the wavelengths to be close to the window size. Hence, frequency resolution changes drastically over the spectrum, and only a small frequency band can be analyzed at the same time. DWT does not have this drawback. It does not rely on a window function. Similar to CWT, it uses wavelets that dilate and contract with frequency to represent the signal. However, in contrast to CWT, it uses far fewer wavelets to represent the signal. This makes DWT a very fast time–frequency estimator. Finally, to complete the time–frequency landscape and allow a thorough comparison on accuracy, we added the high-resolution Wigner–Ville distribution (WVD)^4^, the advanced Hilbert–Huang transform (HHT)^31^ and the more recent empirical wavelet transform (EWT)^32^. WVD has the highest time–frequency resolution mathematically possible and HHT and EWT improve the resolution by using a slow but accurate adaptive iterative process to decompose a signal into fundamental functions that are not necessarily sine functions (for example, FFT). Manual tuning obtained the following parameters for optimal time–frequency sharpness. fCWT and CWT use the complex Morlet wavelet (*σ* = 6) and a frequency scale of 480 frequencies (range, *f*_0_ = 0.25 Hz to *f*_1_ = 250 Hz), evenly spaced in exponential space (cf. the 111Benchmark section). STFT uses a 500-ms Blackman window with 400-ms overlap, DWT uses 11 dyadic (that is, *a*^*j*^ = 2^*j*^) scales of 15-order Daubechie wavelet decomposition, and WVD does not take parameters. HHT and EWT use a frequency resolution of 0.25 Hz. HHT uses seven intrinsic modes that were extracted using a maximum signal-to-residual ratio of 20 as a stopping criterion. EWT decomposes the signal using a peak threshold of 5%. Outliers that deviated more than 3 s.d. from the mean were removed. The HHT had four outliers, which resulted in *N* = 96 for all algorithms to ensure equal group sizes.

**Fig. 4:**
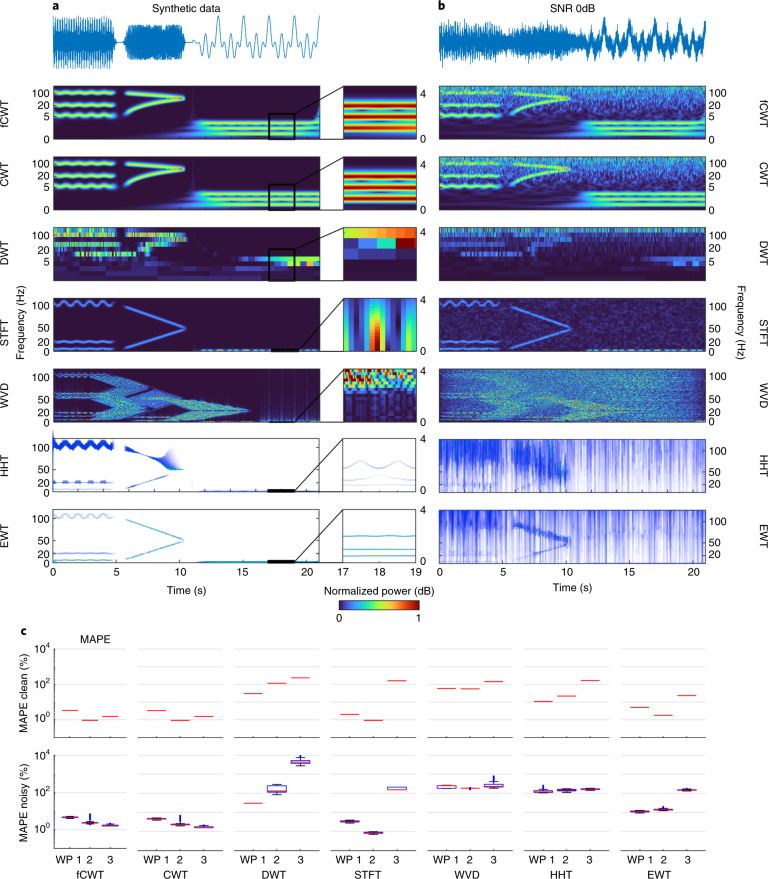
Benchmark results for synthetic data. **a**, Synthetic data composed of wavepackets WP1, WP2 and WP3 (see Methods for details). Seven time–frequency estimation techniques that cover a frequency range from *f*_0_ = 0.25 Hz to the Nyquist frequency *f*_1_ = 250 Hz are shown. fCWT and CWT use the Morlet wavelet (*σ* = 6) and 480 frequencies to divide the spectrum, DWT uses 11 levels of 15-order Debauchie wavelet decomposition, and STFT uses a 500-ms Blackman window with 400-ms overlap to obtain optimal time–frequency resolution. WVD takes no parameters. HHT and EWT have a frequency resolution of 0.25 Hz and rely on an adaptive iterating process. HHT uses seven intrinsic modes that were extracted using a maximum signal-to-residual ratio stopping criterion. A close-up of the time–frequency estimation of the third wavepacket is also shown for comparison. As relative intensity is of primary interest, the spectra are normalized to a [0, 1] range. **b**, As in **a**, but 0-dB white Gaussian noise is added to the synthetic data. The parameters remained the same. **c**, MAPE scores for the clean and noisy data. Boxes show the median and 25th to 75th percentile range; whiskers show minima and maxima. In the top plot only medians are visible as results on the clean dataset are deterministic and, hence, contain no variance. See Supplementary Table 1 for the distribution statistics. Source data

Overall, the per-wavepacket MAPE scores differed significantly on both the clean and noisy datasets between the algorithms (*F*(6, 90) = 112, 243.890, *P* ≪ 0.001, *η*^2^ = 1.000; Fig. 4). Within each algorithm, the per-wavepacket MAPE scores also differed significantly between each other (*F*(2, 94) = 399.044, *P* ≪ 0.001, *η*^2^ = 0.895) However, fCWT and CWT generated similar, low MAPE scores on both the clean and noisy datasets for all three wavepackets. This was confirmed by a correlation analysis per wavepacket, respectively *r*(94) = 0.996, *P* < 0.001, *r*(94) = 1.000, *P* < 0.001 and *r*(94) = 0.997, *P* < 0.001. The low MAPE scores can be explained by CWT’s and fCWT’s wavelet convolution, which averages fluctuations of a signal at different scales^33^, and its redundancy (that is, wavelets are not orthogonal at different scales), which reduces noise by canceling out the random signal components^34^. Hence, both can separate frequency bands and their details across the full frequency range. When compared to the slow CWT, fCWT’s accuracy and noise-handling capabilities are not compromised by its highly efficient implementation. Small differences in the time–frequency spectrum can be seen at the edges. However, these differences are caused by MATLAB’s mitigation of edge artifacts (202020Implementation of fCWT section in the Methods).

STFT cannot extract details of the lower frequency bands present in the first and third wavepackets. The wavelengths of these waves are too long for the 500-ms window we used, whereas a larger window cannot distinguish the complex non-stationary behavior of the first packet. Nevertheless, STFT shows strong noise-handling capabilities that result from the averaging effect of FFT’s inherited convolution. DWT is powerful in denoising, but not suitable for time–frequency analysis. WVD suffers from its well-known artifacts, which are only made worse by the additive noise^4^. HHT and EWT are very good at separating the frequency bands of the clean dataset. Unfortunately, HHT’s frequency estimations, and to a lesser extent those from EWT, fluctuate heavily, leading to high MAPE values. These distortions are caused by the interference between the multiple wavefunctions in each wavepacket. This effect increases dramatically for both algorithms in the noisy dataset^4^.

### EEG

Owing to its ease of measurement and high temporal resolution, the vast majority of neuroscience studies are based on EEG measurements^35^. As EEG measures brain activity via electrodes on the skull, no medical procedures are needed. However, such external measurements do suffer from increased noise. Fluctuations in EEG caused by brain activity are orders of magnitude smaller than the disturbances caused by eye, face and body movements^36^. Therefore, studies average the recordings of many trials to cancel random fluctuations. Unfortunately, the use of repeated trials removes the temporal advantage of EEG and prevents its applicability in real-time implementations, which rely on single-trial estimation.

The often-used FFT cannot handle the highly non-stationary character of EEG signals. Additionally, EEG sampling frequencies are often 1 kHz, and the simultaneous recording of 64 electrodes is standard. Hence, high-speed, non-stationary, time–frequency analysis is essential to have any chance of success in single-trial estimation. This is a criterion that current time–frequency techniques are unable to meet. Techniques like STFT and DWT^8^ are fast but lack the desired resolution in representation, whereas methods like CWT^6^ are precise but lack speed. fCWT fuses the best of both worlds by accelerating the high-resolution CWT by 34 to 122 times. So, we can improve the resolution by ≥34 times or handle ≥34 times as many data than the fastest CWT implementation available in the same time frame. To demonstrate the impact of real-time super-resolution on neuroscience, fCWT was thus benchmarked against full-resolution CWT and fast STFT, and DWT on a single-trial EEG dataset of subjects performing mental arithmetic tasks^37^.

Because active concentration is known to be most visible in the frontal region of the brain^36^, the signals of three frontal electrodes (pre-frontal 1, pre-frontal 2 and mid-frontal in the 10–20 system^36^) were averaged to reduce local fluctuations. We analyzed the resulting signal in the δ (delta), θ (theta), α (alpha), β (beta) and γ (gamma) frequency bands, using a frequency range that spans five octaves (*f*_0_ = 2 Hz to *f*_1_ = 64 Hz). Simultaneous analysis of all these frequency bands is vital for cognitive task experiments, with pre-frontal δ frequencies (2–4 Hz) being associated with attention and motivation^38^, and the power of θ oscillations (4−7 Hz) reflecting memory encoding and retrieval^39^. Lower α-desynchronization (8–13 Hz) relates to task-unspecific attentional demands and β-band (13–30 Hz) power increases with demanding cognitive tasks^36^. The γ oscillations (~30−100 Hz) indicate complex cognitive thinking (for example, object recognition and sensory processing^40^). Consequently, full-range, high-resolution frequency analysis is vital.

**Fig. 5:**
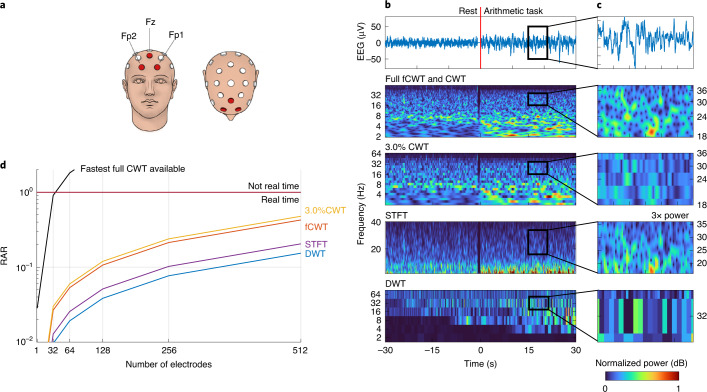
Benchmark results of human EEG data. **a**, The Fp1 and Fp2 pre-frontal and Fz mid-frontal EEG electrodes, which were averaged to assess mental workload. Credit: Imagewriter/Alamy. **b**, Full fCWT and CWT, 3.0%CWT, STFT and DWT of EEG, recorded during 30 s of rest and 30 s of mental arithmetic. Full fCWT and 3.0%CWT analyze the signal using the Morlet wavelet (*σ* = 20) at 650 and 20 scales, evenly spaced in exponential space, respectively. STFT uses a 500-ms Blackman window with 400-ms overlap and DWT uses 11 levels of 15-order Daubechie wavelet decomposition. Spectra are normalized to [0, 1], except for a few spectra that are amplified to enhance visibility. **c**, Zoomed view during the arithmetic task to show each algorithm’s ability to extract the intricate time–frequency details of the β frequency band (13–30 Hz). **d**, The RAR (equation (1)) of full fCWT and CWT, 3.0%CWT, STFT and DWT versus the number of electrodes with a 1-kHz EEG signal. Source data

The analysis of CWT, fCWT, STFT and DWT was complemented with 3.0%CWT (that is, CWT with fCWT’s RAR; Fig. 5). 3.0%CWT enables a fair comparison between the real-time resolution of CWT and full fCWT using 650 frequencies and 3.0%CWT using 20 frequencies. The three CWTs use the complex-valued Morlet wavelet (*σ* = 20), tuned for optimal time–frequency resolution. Based on manual tuning we set a 500-ms Blackman window with 400-ms overlap for STFT and 11 dyadic (that is, *a*^*j*^ = 2^*j*^) scales of 15-order Debauchie wavelet decomposition for DWT, enabling maximal time–frequency sharpness. RAR versus the number of 1-kHz channels was calculated for full-resolution CWT and fCWT, STFT and DWT.

The resolution difference between the equally fast full fCWT and 3.0%CWT is most prominent during the mental arithmetic task. Real-time fCWT distinguishes different EEG frequency bands much better than real-time CWT. The sheer amount of subdivisions in the frequency spectrum allows fCWT to show the small chaotic β-frequency variations often seen during active concentration^36^ and the slow oscillating δ-band power associated with motivation^38^, in real time. Having the same runtime, the fastest CWT implementation fails. Although STFT can separate frequencies in the β-frequency (13–30 Hz) and γ-frequency (~30−100 Hz) bands, it suffers from low spectral resolution in the δ-frequency (<4 Hz) and θ-frequency (4–7 Hz) bands. Hence, STFT makes wide-band EEG analysis impractical. Again, DWT was shown to be unsuitable for detailed time–frequency analysis.

fCWT’s power excels when an entire array of EEG electrodes is analyzed in real time. Although the use of EEG is gaining popularity, its low spatial resolution remains a huge drawback. Figure 5 shows that the fastest CWT implementation available can only handle ~20–24 electrodes (or streams of data) simultaneously at full resolution in real time. By contrast, fCWT is easily capable of calculating real-time, high-resolution time–frequency representations of state-of-the-art EEG set-ups with up to 512 electrodes.

### In vivo electrophysiology

Using depth electrodes, local field potentials (LFPs) measure local voltage changes inside the brain caused by the activity of neuron clusters. LFPs are recorded in vivo and, consequently, they do not suffer from the skull’s high-frequency mask behavior. Consequently, the γ-frequency (~30–100 Hz) and high γ-frequency (>100 Hz) bands can be reliably recorded, these being bands that highly correlate with single neuron firing and reflect aspects of movement (in the motor cortex^41^) and vision (in the visual cortex^42^). Recording these frequencies requires sampling rates that are several times those used for EEGs (that is, 2–3 kHz). Furthermore, in vivo electrophysiology techniques^43^ use huge amounts of electrodes^44^. LFPs are often recorded simultaneously at 100–300 channels, or even more^45^. In the future, data bandwidth is expected to increase even more than its recent tremendous increases. Neuropixels^43^, Utah arrays^44^ and Michigan probes^46^ are currently able to measure hundreds of LFPs and thousands of neurons simultaneously. Real-time LFP time–frequency analysis could lead to next-generation prostatics^41^. Unfortunately, current implementations are unable to handle these bandwidths without compromising resolution. fCWT shows that super-resolution can be maintained when analyzing hundreds of high-bandwidth LFP data streams simultaneously.

Rodent in vivo electrophysiology data from the Allen Brain Observatory data collection^47^ were analyzed. During randomly alternating full-field, high- and low-contrast flashes, six Neuropixel probes^43^ with 374 electrodes (Neuropixel 3a; 20 μm vertical electrode separation) each recorded a mouse visual cortex’s responses. LFPs were obtained by downsampling the data to 1.25 kHz and filtering using a 1,000-Hz low-pass filter. Full fCWT and CWT, 3.0%CWT (EEG section), STFT and DWT time–frequency estimations were performed on 9 s of raw single-trial LFP data containing four stimuli.

We compared CWT and fCWT to STFT and DWT, as the latter two are used in situations where speed is key. Other time–frequency algorithms offer much higher resolution but are orders of magnitude slower, making them impractical for LFP analysis.

The analysis covers a frequency range from *f*_0_ = 8 Hz to *f*_1_ = 128 Hz, allowing simultaneous analysis of both low frequency (that is, α and β bands) and high frequency (that is, γ and high γ bands), which is very important as they reflect different aspects of task performance. Low-frequency LFPs unveil long-distance communication, whereas high-frequency activity reflects local neural processing^48^. As the interplay between these frequency ranges discloses the coordination at the inter- and intra-cortical level^49^, real-time, wide-band time–frequency estimation is key in the LFP analysis of complex brain mechanics.

The three CWTs use the complex-valued Morlet wavelet (*σ* = 16), tuned for optimal time–frequency resolution. Based on manual tuning we set a 500-ms Blackman window with 400-ms overlap for STFT and 11 dyadic (that is, *a*^*j*^ = 2^*j*^) scales of 15-order Debauchie wavelet decomposition for DWT, enabling maximal time–frequency sharpness. The RAR versus number of channels was also calculated for fCWT and CWT at full resolution and STFT and DWT for a 2.5-kHz input signal.

**Fig. 6:**
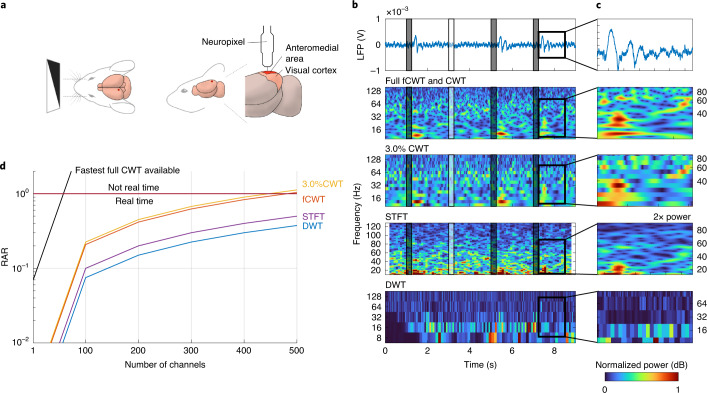
Benchmark results of in vivo electrophysiology data. **a**, In vivo electrophysiology measurements were obtained by the insertion of a Neuropixel^43^ inside the anteromedial area of a rodent’s visual cortex. Mouse drawing adapted from ref. ^84^ under a CC BY license. **b**, Time–frequency estimations by fCWT, CWT, STFT and DWT during 9 s of four 250-ms full-field, high- and low-contrast flashes. The LFP shows exclusive activation after the black stimuli. Full fCWT and 3.0%CWT analyze the signal using the Morlet wavelet (*σ* = 16) at 520 and 16 scales evenly spaced in exponential space, respectively. STFT uses a 500-ms Blackman window with 400-ms overlap and DWT uses 11 levels of 15-order Daubechie wavelet decomposition. Spectra are normalized to [0, 1], except for a few spectra that are amplified to enhance visibility. **c**, Zoom-in of the β- (15–30 Hz), γ- (32–100 Hz) and high γ-frequency bands (>100 Hz), immediately after a black stimulus. Three frequency components in the β-frequency band and two γ bursts are present. Plot scales are aligned as well as possible, despite differences in exponential scale (fCWT and CWT) and linear scale (STFT). **d**, The RAR (equation (1)) of full-resolution fCWT and CWT, 3.0%CWT, STFT and DWT versus the number of channels in a 2.5-kHz electrophysiology signal. Source data

The subfigures of Fig. 6c show the ability of real-time, full fCWT to separate multiple β-frequency components (16, 20 and 25 Hz), locate four γ bursts and reveal the overall γ-frequency dynamics, all at the same time. By contrast, real-time 3.0%CWT misses two out of four γ bursts, cannot separate low-frequency β components, and loses higher γ-frequency dynamics. With STFT, the resolution is on par in the mid-frequency range, but the high- and low-frequency ranges suffer from low resolution. Despite their very high speeds, both STFT and DWT are unsuitable for broadband, high-resolution, time–frequency estimations.

Electrode density is set to increase dramatically; for example, 5,000-electrode Neuropixels have already been announced^50^. Figure 6d shows RAR (equation (1)) versus the number of channels per algorithm. Full CWT can hardly process 15 LFP channels (or data streams) in real time. By contrast, fCWT offers a real-time, full-resolution performance for up to 350–400 channels. Considering the Allen Brain Observatory dataset, fCWT supports real-time analysis and feature extraction of three to four entire Neuropixel probes, whereas the fastest CWT implementation available supports only one-tenth of a single probe.

## Discussion

One of WT’s most powerful features is the possibility to use custom wavelets. However, not all wavelet types are suitable for existing fast approximate CWT implementations, which rely on finite impulse response filters^4^. fCWT does not suffer from this setback, as it calculates wavelets starting directly from its definition. With custom wavelets, fCWT performance can be improved even further^51^. As such, fCWT enables the real-time analysis of high-frequency non-stationary signals, such as in audio^22–24,52^, biosignals (for example, brain–computer interfaces^12^ and ECG^11,13^), image and video^25,26^, sonar and radar^27,28^, renewable energy management^16,17^, cybersecurity^14,15^ and machine fault diagnosis^29,30,53^ (Fig. 1).

The implementation of fCWT could be extended to other time–frequency methods as well. The synchrosqueezed transform (SST)^54^ uses reassignment to sharpen the CWT spectrum, and the chirplet transform (CT)^55^, superlets (SL)^6^ and the noiselet transform (NT)^56^ use atoms to describe a signal, sharing a wavelet-like implementation. Future research could explore speed-ups of these algorithms and bring them to real-time applications. Hence, fCWT’s impact is broader than CWT-based applications alone. Consequently, we did not include the SST, CT, SL and NT in the benchmark study, as these rely on the CWT in their core. These second-order techniques as well as modifications of the included first-order techniques (for example, smoothed WVD^6^) are by definition slower than the already expensive CWT.

fCWT shares its mathematical definition with CWT and, hence, without compromise, inherits both all its benefits^10^ and all its limitations (for example, its degrading spectral resolution^57^ and increasing redundancy in higher frequency ranges^5^). Fortunately, these are well-known limitations that have solutions^4,54^. Moreover, the time–frequency landscape keeps growing, including new CWT implementations^58^. We therefore invite everyone to compare their implementations against fCWT’s open source^59^, and, to extend its validity, we invite all to apply fCWT on more extensive and different specimens that fall outside this paper’s scope.

fCWT allows an acceleration in the developments of science and engineering, industry and health (Fig. 1). Although maintaining CWT’s full resolution and supporting customization, fCWT enables real-time time–frequency analysis of non-stationary signals. As such, fCWT can bring offline research that is hindered by the low resolution of DWT, the limited range of STFT and/or the computational burden of CWT into real-time practice.

## Methods

### Datasets

In this Resource paper, three types of data were used: synthetic, EEG and in vivo electrophysiological data. Details on each dataset are described in the following subsections.

#### Synthetic data

Two synthetic datasets were generated for this paper, both composed of the same three time-varying wavepackets with a sampling frequency of 500 Hz:

1. Three 5-s sine waves, the frequencies of which gradually change between 100 and 110 Hz, 20 and 22 Hz and 5 and 6 Hz, respectively, with a periodicity of 1 Hz.
2. Two 5-s sine waves with linearly changing frequencies between [5, 50] and [100, 50] Hz.
3. Three 10-s low-frequency waves of 2, 1 and 0.5 Hz. All wavepackets are separated by 0.5 s and are multiplied by a Gaussian window function to mitigate discontinuities at the boundaries.

One set contained clean data and the other was contaminated with white Gaussian noise with a 1:1 signal-to-noise ratio (SNR) across the whole signal, with the SNR being determined by the average power. Both datasets have a total duration of 21.0 s and are available in the fCWT CodeOcean repository^59^.

#### EEG

The EEG mental arithmetic dataset by Zyma et al.^37^ was obtained from PhysioNet^60^ and loaded into MATLAB R2021a. EEG data were recorded monopolarly at 500 Hz, using Ag/Ag electrodes and the Neurocom EEG 23-channel system (Ukraine, XAI-MEDICA). The International 10/20 scheme was used for electrode placement. Electrodes were referenced to the interconnected ear reference electrodes. Data were preprocessed using a 30-Hz high-pass filter and a 50-Hz power line notch filter. Common EEG artifacts were removed using independent component analysis. All participants had normal or corrected-to-normal vision and had no mental or cognitive impairment.

In this paper we use the data of subject 13, a 24-year old male who excelled in mental arithmetic by performing 34 subtractions between four-digit and two-digit numbers in 4 min. Subject 13 was chosen to ensure task compliance. We used the last 30 s of EEG during rest and the first 30 s of EEG during the arithmetic task.

#### In vivo electrophysiology

In vivo electrophysiology data were collected from The Visual Coding—Neuropixels project^47^. LFP data from female specimen 738651054 from stimuli IDs 3861−3864 were used. Six Neuropixel version 3a probes were inserted into the mouse visual cortex. In this study, LFP data from fifth probe (Probe ‘e’) channel 63 were used. The 250-ms high-contrast stimuli, 2,000 ms apart, alternate in random order. Mice were shown a neutral gray screen between stimuli. Additional technical, experimental and medical details about the dataset can be found in ref. ^47^.

### Mathematical preliminaries

#### The Fourier transform

With its core idea that a function, often a signal, can always be decomposed into pure sine and cosine functions, the FT is foundational in spectral pattern analysis^3,4,8,61^. However, not all functions *f*(*t*) can be decomposed—only those that live in the Lebesgue space *L*^2^(0, 2π). This space includes all functions that are (1) finite in energy, (2) 2π-periodic and (3) square-integrable, formally

2 $${\int\nolimits_{0}^{2\uppi }| {f(t)}{| }^{2}{{{\rm{d}}}}{t} < \infty \qquad {t}\in {({0},\,{2\uppi })}}$$

3 $${f(t)}={{f}({t}-{2\uppi })}\qquad {t}\in {{{\mathbb{R}}}},$$

which allows *f*(*t*) to be represented as a weighted sum of complex wavefunctions:

4 $${f(t)}={\mathop{\sum }\limits_{-\infty }^{\infty }{c}_{n}{\rm{e}}^{2\uppi int}},$$

with the Fourier coefficients *c*_*n*_ given by the amount of overlap between the conjugated complex wavefunction and the function *f*(*t*):

5 $${c}_{n}={\frac{1}{2\uppi }}{\int\nolimits_{-\infty }^{\infty }{f(t)}{\rm{e}}^{-2\uppi int}}$$

or in discrete form when used on actual digital samples in a sequence *f* having length *N*:

6 $${x}_{k}={\mathop{\sum }\limits_{n=0}^{N-1}{f[n]}{\rm{e}}^{-i2\uppi kn/N}}.$$

In other words, any 2π-periodic, square-integrable function *f*(*t*) can be represented by this superposition of complex-valued sinusoidal waves that are translated in the frequency domain. However, this is precisely Fourier’s pitfall; not all functions, or signals for that matter, are 2π-periodic. FTs cannot decompose the wide variety of non-stationary functions that are not 2π-periodic. Unfortunately, this constraint is often misunderstood, and FT are still used to analyze signals with varying frequencies.

The mathematical reason behind FT’s constraint becomes apparent when we consider the Lebesgue space $L^2({\mathbb{R}})$ containing all square-integrable functions that have finite energy along the entire real axis:

7 $${\int\nolimits_{-\infty }^{\infty }| {f(t)}{| }^{2}{{{\rm{d}}}}{t} < \infty }.$$

The reason why equation (4) cannot represent these functions is that pure sine waves extend to infinity and therefore do not have finite energy. Pure waves do not lie in $L^2({\mathbb{R}})$ and, as such, they cannot represent its functions.

#### Wavelets

We can define a set of functions other than equation (4) that do have finite energy. The result is the set of short periodic functions *ψ*(*t*) called wavelets that are well localized in both the time and frequency domains^5,6,8,33,57,62^. Consequently, wavelets need to be able to translate in both domains as well:

8 $${\psi }_{jk}{(t)}={2}^{-j/2}{\psi }({2}^{j}{t}-{k}),$$

where *ψ*_*jk*_ is a daughter wavelet function, defined as the mother wavelet *ψ*(*t*) scaled in the frequency domain by *j* and translated in the time domain by *k*. So, the WT outputs a 2D time–frequency matrix, where the FT gives a 1D frequency spectrum.

Similar to equation (4), the superposition of these wavelets can represent any function

9 $${f(t)}={\mathop{\sum }\limits_{{j,\,k} = {-\infty }}^{\infty }{c}_{jk}{\psi }_{jk}{(t)}},$$

where, like with the FT, the wavelet coefficients *c*_*jk*_ are given by the amount of overlap between the wavelet and the function *f*(*t*). This definition also shows us that wavelets, similar to Fourier’s pure wavefunctions, live in Hilbert space as multiplication between two functions is defined as an inner product:

10 $${W}_{\psi }{f(j,\,k)}={\langle {f},\,{\psi }_{jk}\rangle }={{c}_{jk}}={\int\nolimits_{-\infty }^{\infty }{f(t)}{\overline{\psi }}_{jk}{(t)}{{{\rm{d}}}}{t}},$$

in which ${\overline{\psi }}_{jk}$ corresponds to the conjugate of *ψ*_*jk*_. However, as *j* and *k* can be any real number, we have to define both variables’ optimal discretization such that the resulting time–frequency matrix does not under- or overdetermine the function *f*(*t*). So, the variables should be discretized such that the wavelets form an orthogonal basis in Hilbert space^63,64^—in other words, such that the wavelet functions have zero overlap.

Wavelets are orthogonal in Hilbert space if

11 $${{\langle {\psi }_{jk},{\psi }_{lm}\rangle }={\delta }_{jk}{\delta }_{lm}},$$

from which it follows that equation (8) is indeed logarithmic orthogonal. The WT that uses this type of discretization is called the DWT^8,65,66^. In this context, ‘discrete’ refers to the use of its wavelets, not to the type of data it processes. As all DWT’s wavelets are orthogonal, it describes a function by the minimal number of wavelet coefficients possible. However, as stated at the beginning of this paper, a redundant, overcomplete representation is often much more favorable for signal analysis. Therefore, it is also possible to define a WT with arbitrary wavelet discretization. Such a wavelet transformation is called the CWT^67^. Again, ‘continuous’ does not refer to the type of data it can handle. CWT features continuously scalable and translatable wavelets that allow a much more precise analysis of a signal’s spectrum:

12 $${W}_{\psi }{f(a,b)}={| }{a}{| }^{-1}{\int\nolimits_{-\infty }^{\infty }}{f(t)}{\overline{\psi }}{\left(\frac{{t}-{b}}{a}\right)}{{{\rm{d}}}}{t},$$

which comes with considerable computational complexity. When implemented digitally, its discrete form is used:

13 $${W}_{\psi }{f[a,b]}={| }{a}{| }^{-1}\mathop{\sum }\limits_{{n}={0}}^{{N}-{1}}{f[n]}{\overline{\psi }}{\left[\frac{{n}-{b}}{a}\right]},$$

which is mathematically equivalent to passing the input signal through a series of wavelet filters of different lengths. Care is required at the boundaries of the signal. As the discrete form assumes signals of finite length, wavelet coefficients near the boundaries become increasingly meaningless. Instantaneous frequency at the first or last sample is impossible to calculate as one should know how the signal continues. There are several strategies to solve this uncertainty. For more details about this topic, see the Boundary effects section.

Equation (10)’s computational complexity can be estimated using the trapezoidal rule for integral solving and assuming a signal of length *N* = 2^*J*^. Furthermore, we assume *J* wavelets at *a*_*j*_ = 2^*j*^ discrete scales, and a wavelet length of *L* samples at unit scale. Starting at unit scale *a*_0_ = 1, we then have *O*(*a*_0_*NL*) complexity, with the cost of all scales resulting in

14 $${NL} + {2NL} + {4NL} + {\ldots} + {{2}^{J}}{NL} = {O}{({L}{{N}^{2}})}.$$

In other words, a naïve approach to DWT calculation would result in a polynomial complexity of *O*(*N*^2^). CWT would be even worse, as the discretization of the time and frequency domains is much finer. Fortunately, scientists quickly realized a considerable reduction in computational complexity could be achieved using Parseval’s theorem.

#### Fourier-based wavelet transform

Applying Parseval’s theorem to equation (12), a reduction in CWT’s complexity can be achieved:

15 $${W}_{\psi }{f(a,\,b)}={\frac{1}{2\uppi }}{\int }{\hat{f}}{(\xi )}{\overline{\widehat{{\psi }_{a,\,b}}{(\xi )}}}{{{\rm{d}}}}{\xi }.$$

Subsequently, we define ${\overline{\widehat{{\psi }_{a,\,b}}{(\xi )}}}$ in terms of the FT of the mother wavelet function *ψ*(*t*), using its basic time-shifting and time-scaling properties:

16 $${\widehat{{\psi }_{a,\,b}}}{(\xi )}={\frac{1}{a}}{\hat{\psi }}{(\xi )}{\rm{e}}^{-ib\xi }\qquad ({{\rm{time}}\,{\rm{shifting}}})$$

17 $${= {\hat{\psi }}{(a\xi )}{\rm{e}}^{-ib\xi }\qquad ({{\rm{time}}\,{\rm{scaling}}})}.$$

Substitution gives

18 $${W}_{\psi }{f(a,\,b)} = {\frac{1}{2\uppi }}{\int }{\hat{f}}{(\xi )}{\overline{\hat{\psi }{(a\xi )}}}{\rm{e}}^{ib\xi }{{{\rm{d}}}}{\xi }$$

or in its discrete form

19 $${W}_{\psi }{f[a,\,b]}={\frac{1}{K}}{\mathop{\sum }\limits_{k=0}^{K-1}}{\hat{f}}{[k]}{\overline{\hat{\psi }[ak]}}{\rm{e}}^{{i2\uppi bk}/{K}},$$

which describes *W*_*ψ*_*f*[*a*, *b*] as an inverse FT of ${{\hat{f}}{[k]}{\overline{\hat{\psi }{[ak]}}}}$. So, WT’s computational complexity no longer depends on the time-offset parameter *b*. As ${\hat{f}{[k]}}$ can be calculated beforehand, it is reduced to three distinct steps per scale:

1. Generate $${\overline{\hat{\psi }{[ak]}}}$$
2. Calculate $${{\hat{f}}{[k]}{\overline{\hat{\psi }{[ak]}}}}$$ and
3. Evaluate the inverse FT and obtain *W*_*ψ*_*f*[*a*, *b*],

with the first two steps evaluated in *O*(*N*) and the last one requiring at least *O*(*N*log_2_*N*) when using a fast FT implementation^68,69^. This results in *O*(*N*log_2_*N*) complexity, a considerable reduction compared to *O*(*N*^2^), which is needed for the naïve approach. Additionally, the constant factor of this complexity can be reduced even more, as we will see in the next section.

#### Implementation of fCWT

Fourier-based wavelet transformation’s computational complexity is mainly determined by the inverse FT. Consequently, equation (12) has been rewritten regularly to use spline interpolation of the wavelet and circumvent the FT entirely^70,71^. Spline interpolation, also known as polynomial interpolation, defines a wavelet by only a few evenly spaced sampling points across the domain. Because the number of points is independent of the wavelet’s scale, the theoretical complexity of equation (12) is reduced to linear time. However, while complexity is lowered, the constant factor that equals the number of sampling points has been increased tremendously. In turn, this yields a trade-off between speed and accuracy: more interpolation points leads to increases in both precision and computation time. Additionally, the spline interpolation only works for specific wavelet types. To avoid the trade-off, we optimize the Fourier-based wavelet transformation by reducing the constant factor of its computational complexity. In this way, we maintain WT’s ability to use custom wavelet types^51^ and can exploit optimized FFT libraries^72–74^.

**Extended Data Fig. 1:**
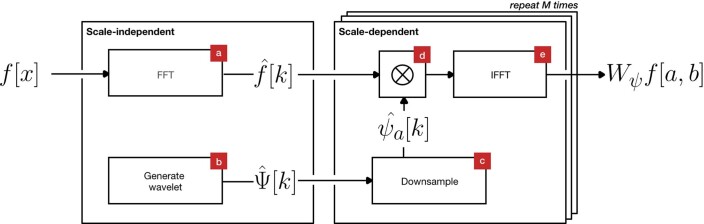
Algorithmic implementation of fCWT The algorithmic implementation behind fCWT can be divided into: i) scale-independent and ii) scale-dependent operations. The scale-dependent operations each calculate the wavelet coefficients of a single scale-factor in the final time–frequency matrix. By repeating the scale-dependent part *m* = ∣*a*∣ times, the time–frequency matrix is build up one row at a time.

fCWT separates scale-independent and scale-dependent operations, which have to be performed separately for each wavelet’s scale. A detailed schematic of fCWT’s algorithmic implementation is provided in Extended Data Fig. 1. With CWTs, the frequency scale is often divided into hundreds of scales. We thus focused the optimization on the fCWT’s scale-dependent part by exploiting its repeated nature and high parallelizability. The scale-independent operations are performed first as their result forms the input for the scale-dependent steps. We pre-calculate two functions: (1) the input signal’s FFT and (2) the FFT of the mother wavelet function at scale *a*_0_ = 2. Both functions are independent of the scale factor *a*, so they can be pre-calculated and used as look-up tables in the processing pipeline.

##### FFT

Using the float- and AVX2-enabled Fastest Fourier Transform in the West (FFTW) library^73^, the input signal’s FFT is calculated. FFTW has superior performance in various benchmarks^75^ and has the ability to dynamically optimize its algorithmic implementation. FFTW determines the most efficient way to calculate the signal’s FFT with length *N* on hardware set-up *X*. This requires considerable time, which makes it only useful in situations where many FFTs are calculated with the same *N* and *X*. This is the case with fCWT, as its scale-dependent part evaluates a fixed-length inverse FFT for every scale factor *a*. Other high-performance FFT libraries include the Fastest Fourier Transform in the South^72^ and Intel’s Math Kernal Library^74^. However, as Fastest Fourier Transform in the South lacks important optimization techniques and Intel’s Math Kernel Library is limited to Intel processors only, FFTW is currently the most flexible and versatile high-performance FFT library available.

**Extended Data Fig. 2:**
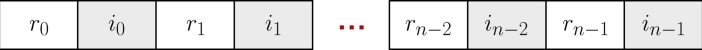
FFTW’s interleaving storing format Using an interleaving value format, the Fastest Fourier Transform in the West (FFTW) writes a complex-valued Fourier transform to memory. As the CPU caches adjacent values when accessing memory, accessing the complex and real part only requires single memory access instead of two.

Before a signal’s FFT is calculated, it is first zero-padded to the nearest power of two, which allows more time-efficient calculations than with other signal lengths. Zero padding lets all signals that map to the same nearest power of two use the same FFTW optimization. Hence, the flexibilty of fCWT as a tool is preserved while still enjoying the benefit of FFTW’s optimization plans. However, it will result in step-like performance behavior as seen in Fig. 3. After FFT calculation, we let FFTW write the complex-valued FT to memory in an interleaving format (Extended Data Fig. 2). Using this, we exploit the CPU’s predictive caching behavior and hence reduce memory access in the next steps. Because a CPU works with chunks of memory instead of single values, it always caches adjacent memory next to a requested value as well^26,76^. While we access the real part of a value, interleaving takes advantage of this behavior as the complex part is cached. Consequently, accessing the complex part after the real part does not require an additional memory request, which reduces memory accesses by 50%.

##### Scale-independent mother wavelet generation

The FFT of the mother wavelet function ${\hat{{{\varPsi }}}}{[k]}$ is generated once during the scale-independent step. Because wavelets in the frequency domain uniformly contract as their scale increases, daughter wavelet functions can be generated by downsampling a pre-generated mother wavelet function. Because scales must be at least *a*_min_ = 2, we generate the mother wavelet function at *a*_0_ = 2 to save memory. It is important to note that the mother wavelet function is generated directly from its analytical Fourier-transformed definition. Consequently, we create ${\hat{{{\varPsi }}}}{[k]}$ such that its length always matches that of ${\hat{f}}{[k]}$. This ensures fCWT’s independence of wavelet length and achieves the highest wavelet resolution possible.

After calculation of the FFT signal and the generation of the FFT mother wavelet, the scale-independent step is complete. fCWT proceeds to the scale-dependent phase (Extended Data Fig. 1). This phase is repeated *m* = ∣*a*∣ times. Using *m* steps with step size Δ*a*, the scale factors *a* are defined by discretizing the frequency spectrum evenly on a logarithmic scale:

20 $${a} = {\{{2}^{x{{\Delta }}a}| {x} \in {N} \wedge {0} < {x} \le {m}\}}.$$

This generates the wavelet coefficient matrix *W*_*ψ*_*f*[*a*, *b*] one row at a time.

##### Scale-dependent downsampling

**Extended Data Fig. 3:**
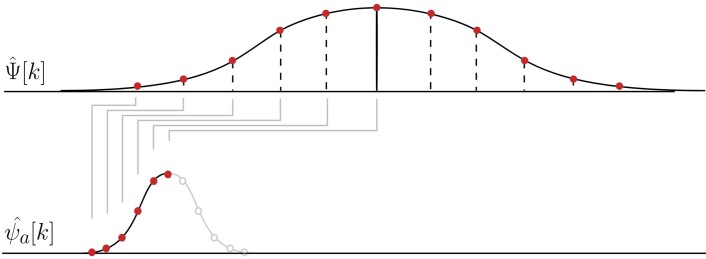
From mother to daughter wavelet The generation of the daughter wavelet ${\hat{\psi }}_{a}[k]$ is done efficiently by downsampling the mother wavelet $\hat{\Psi }[k]$. This eliminates the need for expensive Gaussian calculations in the scale-dependent step. The mother wavelet is only calculated once in the scale-independent step.

Each iteration of the scale-dependent step first generates the Fourier-transformed daughter wavelet function ${\hat{\psi }}_{a}{[k]}$ by downsampling the mother wavelet function generated in the scale-independent step. This optimization is realized by using the mother wavelet as a look-up table (Extended Data Fig. 3). Hence, as explained earlier, the expensive Gaussian calculations involved in wavelet calculation are removed from the scale-dependent step. The daughter wavelet is generated by only performing a systematic look-up that accesses every *a*th value of the mother wavelet function. A schematic overview of this process is shown in Extended Data Fig. 3.

##### Scale-independent multiplication

**Extended Data Fig. 4:**
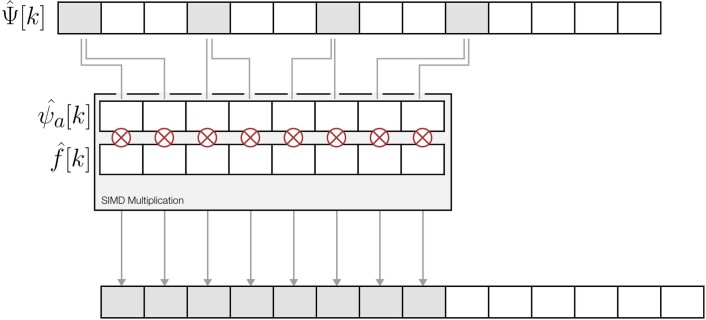
SIMD multiplication fCWT combines the generation of the daughter wavelet and its multiplication with the Fourier transformed input signal together in one Single Instruction, Multiple Data (SIMD) multiplication. As the Fourier transformed input signal is complex-valued, the real daughter wavelet values are copied twice such that SIMD can perform an element-wise multiplication between both buffers. In this example a scale-factor of *a* = 3 is used.

Using the single instruction, multiple data (SIMD) model, another acceleration is achieved. By using the CPU’s full power, eight multiplications are executed at once^77,78^, which is used to exploit the elemental-wise multiplication between ${\hat{\psi }}_{a}{[k]}$ and ${\hat{f}{[k]}}$. In our case, SIMD performs four complex-valued multiplications in parallel, because the multiplication between the real-valued daughter wavelet and complex-valued Fourier-transformed input signal takes two multiplications per element. By exploiting the parallelizable nature of this step, an additional speed-up of 4× is achieved. Extended Data Fig. 4 shows this process graphically.

An additional acceleration is achieved by merging the generation of the daughter wavelet (Scale-dependent downsampling section) and the multiplication with ${\hat{f}{[k]}}$ (Scale-independent multiplication section) in one loop. Consequently, no intermediate results are stored in memory, which largly eliminates memory access.

##### Scale-independent inverse FFT

Finally, using FFTW’s inverse FFT function, the result is transferred back to the time domain. Similar to the FFT calculation in the scale-independent step, the inverse FFT uses a pre-calculated optimization based on the input signal’s zero-padded length. The complex-valued time–frequency matrix is stored in row-major order as an array of 2*NM* floats, where *N* is the signal length and *M* the number of scales. Each value is stored as two floats as the matrix is complex-valued.

##### Boundary effects

Because CWT uses convolution to calculate the wavelet coefficients, a wavelet is eventually close enough to the beginning or end of the signal to be multiplied with undefined data outside the boundaries of the signal. In these situations, frequency becomes a meaningless construct as one does not know how a signal would proceed beyond these limits. As this effect becomes more evident with larger wavelets (that is, lower frequencies) one can speak of a cone of influence^33^ caused by the edges that affect the entire spectrum. Several strategies exist to handle these so-called boundary effects^4,79,80^.

One could extend a signal by adding zeros at the beginning and the end to define data outside the boundaries. Because convolution relies on the element-wise multiplication between the signal and the wavelet, this strategy is similar to stopping the convolution at the boundary. Other strategies rely on making assumptions about the signal outside its bounds. For example, the signal could be extended by mirroring or repeating the signal at its boundaries^4^. With fCWT, we decided to let the users decide themselves. As fCWT is designed to be independent of signal content, we assume an unbiased zero extension. Consequently, users can choose their own boundary strategy by extending the signal manually before the fCWT is applied.

**Extended Data Fig. 5:**
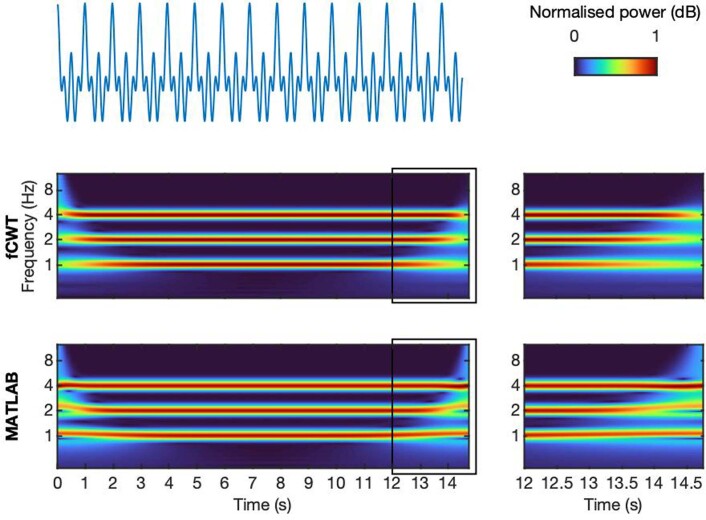
Boundary effects in fCWT and MATLAB With fCWT we perform zero extension to mitigate boundary effects. In contrast, by default MATLAB uses a content dependent mirror extension. In some cases, such an extension strategy can increase boundary effect severity instead of decreasing it as can be seen here. Source data

The direct result of fCWT’s strategy can be seen in the Synthetic data section. At both edges, fCWT shows a strong cone of influence effect of the boundary. MATLAB, by default, performs signal extension, which mitigates these artifacts. However, MATLAB’s default extension strategy sometimes leads to an increase in artifacts instead of a reduction. An extreme example can be seen in a visual comparison between both techniques in Extended Data Fig. 5. Consequently, with fCWT we went for an unbiased zero extension strategy aiming for maximal transparency and flexibility.

#### Time–frequency ridge extraction

To perform quantitative assessment of time–frequency spectra on the synthetic data, a time–frequency ridge extraction methodology is used. This allows a comparison between the ridges (that is, frequency components) in the time–frequency spectra and the actual frequency components used to generate the dataset.

The synthetic dataset (see the Data availability statement for details) consists of three distinct wavepackets. The time–frequency ridge extraction is performed on each wavepacket separately. Each segment is defined such that it trims the first 0.5 s and last 0.5 s of each wavepacket to remove the Gaussian window function influence. In the third wavepacket, 3.0 s is trimmed from the end to remove the influence of boundary effects (Boundary effects section).

MATLAB’s tfridge() is used to extract, respectively, three, two and three ridges from the first, second and third wavepacket, as it is the most used approach to ridge extraction. To do so, tfridge() needs pre-defined penalty coefficients (P_coef_), which determine the stability of the ridge estimation. As wavepackets and time–frequency algorithms largely differ in their characteristics, these penalty coefficients need to be optimized manually for each combination. Manual optimization is performed greedy by first testing different orders of magnitude (P_mag_): *P*_mag_ ∈ {10^−3^, 10^−2^, 10^−1^, 10^0^, 10^1^, 10^2^, 10^3^}. When the optimal magnitude range [*P*_mag,__1_, *P*_mag,2_] is selected, ten equally distanced coefficients are tested within that range *P*_coef_ ∈ *P*_mag,1_ ⋅ {0, 1, 2, …, 8, 9}. The resulting penalty coefficient is chosen for the benchmark. All penalty coefficients are provided in the source data for Fig. 4.

A fair comparison among the algorithms was secured, as the same stable ridge extraction was applied on all included algorithms. However, in future work, alternate open-source algorithms could be worth exploring^81^. These might yield highly accurate ridge extractions and/or remove the need for manually tuned parameters. As such, this might result in an even more fine-grained comparison among the algorithms.

### Supplementary information

Supplementary Table 1 Detailed statistical descriptions of MAPE error distributions as plotted in Fig. 4. Results are obtained from 100 runs of seven time–frequency methods on a synthetic dataset consisting of three wavepackets with SNR = 0 dB Gaussian noise added.

### Source data

Figure 2 source data Zipped example and heatmap source data.

Figure 3 source data Statistical and experimental source data.

Figure 4 source data Zipped statistical and heatmap source data.

Figure 5 source data Zipped experimental and heatmap source data.

Figure 6 source data Zipped experimental and heatmap source data.

Source Data Extended Data Fig. 5 Zipped example and heatmap source data.

## Data Availability

The generated synthetic dataset used in Fig. 4 is provided under ‘data’ in the CodeOcean fCWT capsule^59^. The ‘EEG During Mental Arithmetic Tasks v1.0.0’ used in Fig. 5 is available at https://physionet.org/content/eegmat/1.0.0/. The in vivo electrophysiology data collected by The Visual Coding—Neuropixels project^47^ and used in Fig. 6 is available in the Neurodata Without Borders (NWB) format via AllenSDK (https://allensdk.readthedocs.io). An example Jupyter Notebook for accessing the LFP data is available at https://allensdk.readthedocs.io/en/latest/_static/examples/nb/ecephys_lfp_analysis.html. Source data are provided with this paper.

## Code availability

fCWT is released under Apache License Version 2.0 and will be maintained in a public GitHub repository available at https://github.com/fastlib/fCWT. fCWT is available for Mac OSX, Linux and Windows systems and features a detailed description on how to obtain Visual Studio project-files and generate cpp-based MEX-packages for easy MATLAB integration. For benchmark reproducibility, a version of fCWT at the time of publication is available on CodeOcean^59^.
